# Evidence of alphaherpesvirus infections in Alaskan caribou and reindeer

**DOI:** 10.1186/1746-6148-8-5

**Published:** 2012-01-14

**Authors:** Alina L Evans, Carlos G das Neves, Greg F Finstad, Kimberlee B Beckmen, Eystein Skjerve, Ingebjørg H Nymo, Morten Tryland

**Affiliations:** 1 Section of Arctic Veterinary Medicine, Norwegian School of Veterinary Science, Stakkevollveien 23, N-9010 Tromsø, Norway; 2 Department of Forestry and Wildlife Management, Hedmark University College, Campus Evenstad NO-2418 Elverum, Norway; 3 University of Alaska Fairbanks, Reindeer Research Program. Box 757200, Fairbanks Alaska 99775. USA; 4 Alaska Department of Fish & Game, Division of Wildlife Conservation, 1300 College Road, Fairbanks AK 99701. USA; 5 Centre for Epidemiology and Biostatistics, Norwegian School of Veterinary Science, P.O.Box 8146, NO-0033 Oslo, Norway

**Keywords:** caribou, epidemiology, herd health, herpesvirus infectious diseases, reindeer, *Rangifer*, wildlife medicine

## Abstract

**Background:**

The reindeer (*Rangifer tarandus tarandus*) industry in Alaska began with animals imported from Siberia (Russia) in the 1890's. Cervid herpes virus 2 (CvHV2) is endemic in reindeer in Scandinavia. We sought to determine if the same virus, or similar herpesviruses, were circulating in Alaskan reindeer and caribou (*Rangifer tarandus granti*). Serum samples from 292 reindeer were collected during annual reindeer handlings (1988-2005) near Nome, Alaska. In 2005, swab samples were collected from 40 calves from this herd, near Nome, Alaska. In 2007, ocular and nasal swab samples were collected from 30 apparently healthy reindeer calves near Wales, Alaska. Samples of plasma and white blood cells were collected from three Alaskan caribou herds, Mulchatna (n = 24), Teshekpuk (n = 34) and the Western Arctic (n = 87) in 2009.

**Results:**

Of 292 reindeer samples tested by ELISA for antibodies against alphaherpesvirus (bovine herpesvirus 1 as antigen), seroprevalence was 47% (136/292) and adult reindeer had higher seroprevalence than yearlings. The overall seroprevalence for caribou was 60% (87/145), with no significant differences among caribou herds. A virus neutralization test of 20 samples from both reindeer and caribou showed that ELISA positive samples always neutralized CvHV2 to a greater extent than BoHV1 or elk herpesvirus (ElkHV), indicating that CvHv2 is the most likely virus circulating. PCR of nasal and ocular swabs sampled from 30 reindeer calves in Wales, Alaska (2007) yielded four CvHV2 positive samples. PCR amplicons of the expected size (294 bp) were obtained from 2 of the 36 buffy coats samples from caribou, and the amplicon sequences were consistent with CvHV2.

**Conclusions:**

This study shows that Alaskan reindeer and Caribou are infected with an alphaherpesvirus. Based on sequence similarity, CvHV-2 is the most likely virus. Further studies should be conducted to determine the impact of this infection on the health of these animals.

## Background

Indigenous Rangifer migrated to Alaska more than 13,000 years ago [1], and have been separated from Siberian reindeer since the disappearance of the Bering land bridge 7000 years ago. Semi-domesticated reindeer (*Rangifer tarandus tarandus*) were introduced to Alaska in 1891 when 16 Siberian reindeer were introduced to Unalaska and Amaknak Islands [2]. Over the next 11 years, a total of 1,280 reindeer were imported to western Alaska [3]. Reindeer numbers in Alaska peaked in the 1930's with a population estimate of about 640,000 animals [3]. Under current management, reindeer herds range freely on large designated grazing allotments and are periodically herded into handling facilities for ear tagging, censusing, husbandry and veterinary care [4]. In the last 15 years, the herds have drastically declined in size and many herds have completely disappeared. Most of Alaska's remaining 15,000 reindeer are now found on the Seward Peninsula and Nunivak, St Lawrence and Umnak Islands [5].

There are a variety of possible reasons for this population's decline including commingling and emigration into the migratory Western Arctic caribou herd (WAH, *Rangifer tarandus granti*) and increasing predation by wolves. The WAH is the largest caribou herd in Alaska, with a population of 377,000 in 2007 [6]. Since 1996, this herd has expanded their seasonal migratory routes to include the range of Alaskan reindeer on the eastern half of Seward Peninsula where caribou had been absent for over 150 years [6]. Many reindeer have out-migrated with the caribou during spring migrations, reducing reindeer numbers. Although alphaherpesviruses were previously shown to be endemic in Alaskan caribou [4], it was unknown which virus was present, if this virus was introduced to Alaska with reindeer from either Siberia or Norway and if cross-transmission occurs between reindeer and caribou in Alaska.

Members of the subfamily, *Alphaherpesvirinae *(family *Herpesviridae *order *Herpesvirales*) have a broad host range, replicate quickly, lyse infected cells and establish latency in sensory neuron ganglia. In a range of ruminant species, clinical signs may be present in the eyes, respiratory tract, genital organs, and mammary glands. Systemic spread can result in abortions and enteritis, usually achieved by invasion of the reticuloendothelial system [7].

Alphaherpesviruses antigenically similar to BoHV1 have been found to be endemic in reindeer in Norway [8], Sweden [9], and Finland [10], as well as in caribou in Canada [11] and Alaska [4]. In Finland, a virus isolated in 1982 was later shown to be genetically different from BoHV1 and is now called Cervid herpesvirus 2 (CvHV2) [12].

A recent serosurvey, based on a commercial ELISA kit for cattle and BoHV1, complemented by a virus neutralization test (VNT) showed that CvHV2 is endemic in Norwegian reindeer, with a mean seroprevalence across northern Norway of 48% (1502/3062) [13]. The presence of CvHV2 was also confirmed by virus isolation after reactivation of latent infection with dexamethasone in an experimental infection trial [14].

The clinical impact of CvHV2 infections in reindeer is unclear. Recently, CvHV2 was associated with an outbreak of infectious keratoconjunctivitis (IKC) in Norwegian semi-domesticated reindeer [15]. Of the sampled animals, clinically affected individuals had a CvHV2 seroprevalence of 86% (n = 28), while the prevalence in unaffected reindeer was 42% (n = 12). Although bacteria including *Moraxella *sp., *Streptococcus *spp., *Staphylococcus *spp. and *Archanobacterium *spp. were also isolated, it was concluded that CvHV2 was the primary agent during this outbreak, facilitating secondary bacterial infection. This conclusion was supported by the fact that virus could be isolated from eye swab samples more frequently in animals with a mild stage of the disease, whereas in severe cases of IKC, only bacteria were recovered [15]. However, it remains unknown if the IKC frequently reported in Alaskan reindeer is related to a herpesvirus infection [16].

CvHV2 has been isolated from respiratory and genital swab samples as well as fetal and ocular tissues of reindeer in Norway [14,15,17]. Clinical signs of CvHV2 seen during an experimental infection study demonstrated that respiratory inoculation leads to moderate fever, slight serous nasal discharge, and minor erosions and erythema in the nasal cavity, while genital inoculation leads to moderate fever, vaginal discharge, and erosions of the vulva [14].

A study in Saskatchewan, Canada, found 55% of 42 woodland caribou (*Rangifer tarandus caribou*) to have antibodies to a virus related to BoHV1 [18]. Similar results have also been reported in Alaskan reindeer some decades ago [4], indicating that either BoHV1 or another closely related alphaherpesvirus with similar antigenicity was present. A survey of Alaskan caribou found BoHV1 related antibodies in sera of 6/67 (9%) animals [19].

Persistent viral infections such as those caused by cervid herpesviruses may affect calf mortality and fitness, and potentially result in abortions and weak offspring [20] as also reported in BoHV1 infections in cattle [7].

Our objectives were to 1) describe the level of exposure to herpesviruses in Alaskan reindeer and caribou and to identify risk factors for exposure, 2) determine which herpesvirus is circulating among Alaskan reindeer and caribou and 3) characterize the virus through sequencing of PCR amplicons obtained from nasal and ocular swabs.

## Results

### Reindeer

**Figure 1:**
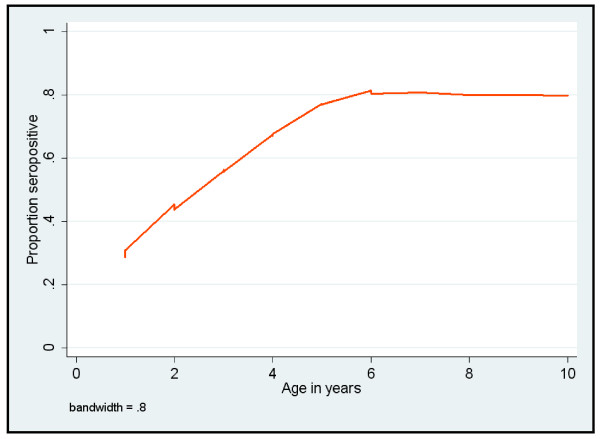
**Locally weighted scatterplot smoothing curve showing the association between the proportion of seropositive animals (alphaherpesvirus) and age of semi-domesticated reindeer (*Rangifer tarandus tarandus*) from Alaska**.

The overall frequency of seropositivity (ELISA) for reindeer was 47% (136/292), showing no significant difference between years of sampling. Two samples having inconclusive results were classified as negative prior to the statistical analysis. Adults had a higher likelihood of exposure (being seropositive) than calves (odds ratio = 4.17; CI 95% = [2.42-7.17]), whereas gender and year of sampling were not significantly associated with seroprevalence (P > 0.05). The area under the ROC curve was acceptable at 0.687. The lowess curve (Figure 1) presents the age trends in reindeer showing an increase in seroprevalence for older animals.

PCR amplicons of the expected size (294 bp) were obtained from 5 of the 50 swabs collected from apparently healthy reindeer calves (4 from the Wales area, 1 from Davis). Sequences were found to be identical to the CvHV2 Salla82 strain (AF078727.2), except for the Davis reindeer sample, which was similar to the Norwegian reindeer isolates of CvHV2 [17], having one single nucleotide synonymous mutation compared to the Salla82 strain. Sequences submitted to GenBank (From Wales, BankIt1453414 Seq1 JF951964, BankIt1453414 Seq2 JF951965, BankIt1453414 Seq3 JF951966, BankIt1453414 Seq4 JF951967, and from Davis, BankIt1453414 Seq5 JF951968).

From the subpanel of samples chosen for the virus neutralization test (VNT), all samples considered negative by ELISA were negative for all viruses in the VNT (2 caribou and 2 reindeer). All samples considered positive by ELISA, except one weak positive (60.8% competition), were positive for at least one virus in the VNT. All samples classified as positive in the VNT neutralized CvHV2 to a higher titer than any other viruses. For reindeer, the highest serum dilution that neutralized CvHV2 was 1:362 followed by ElkHV with 1:181, then CvHV1 with 1:128 and finally BoHV1 with 1:91 (Table 1). Virus back titrations for the reindeer and caribou neutralizations oscillated between 90 and 115 TCID_50_, (90 for CvHV1, 95 for BoHV1, 110 for ElkHV and 115 for CvHV2), a range considered to be narrow enough to compare viruses' neutralizations results.

**Table 1:** Virus neutralization test (VNT) results on samples from reindeer *(Rangifer tarandus tarandus) *and caribou *(Rangifer tarandus granti) *from Alaska.

	Caribou	Reindeer
	***Mean ED_50 _titre^1^***	***Mean ED_50 _titre^1^***

BoHV1	9,4	31,0

CvHV1	13,0	29,3

CvHV2	131,0	136,7

ElkHV1	31,0	55,1

Neutralizing titers were calculated according to the Spearman-Kärber method as the serum dilution necessary to neutralize the cytopathic effect in 50% of the wells (effective dose 50%; ED_50_) and are presented as the mean values for each virus titration (samples classified as negative in ELISA are excluded). Virus back titrations for the reindeer and caribou neutralizations oscillated between 90 and 115 TCID_50_, (90 for CvHV1, 95 for BoHV1, 110 for ElkHV and 115 for CvHV2).

### Caribou

The overall frequency of seropositivity on ELISA for caribou was 60% (87/145) with similar levels in Mulchatna (58%), Teshekpuk (67%), and Western Arctic (57%). The logistic regression model showed that differences in seroprevalence between genders and herds were not statistically significant (P > 0.05). As most animals were calves (140 out of 145) it was not possible to assess the effect of age on seroprevalence.

PCR amplicons of the expected size (294 bp) were obtained from 2 of the 36 buffy coats samples. The amplicon sequences were identical to corresponding sequences of the CvHV2 Finnish strain (Salla-82AF078727.2). Sequences submitted to GenBank (BankIt1453414 Seq6 JF951969 and BankIt1453414 Seq7 JF951970-Caribou 109754).

In the VNT for caribou, the highest serum dilution that neutralized CvHV2 was 1:362 followed by ElkHV with 1:91 then CvHV1 and BoHV1 both with 1:45 (Table 1).

## Discussion

The overall frequency of seropositivity of 60% in caribou and 47% in reindeer with no significant change over a time frame of 16 years for the latter, together with the fact that herpesviruses undergo latency and life-long infection, strongly indicates that an alphaherpesvirus is endemic in these animal populations. As other alphaherpesviruses cross-react serologically to BoHV1 [13], VNT and PCR were required to determine which virus was circulating in Alaskan reindeer and caribou. While VNT titer differences between the viruses may not be considered as great, all samples neutralized CvHV2 to a higher extent than other viruses. Likewise, viral DNA sequencing showed, in a highly conserved gene such as UL27 coding for glycoprotein B, a 99-100% homology to CvHV2, with lower degrees of homology for other ruminant alphaherpesviruses. Based on the gB amplicon sequences, widely accepted to be sufficient to differentiate between ruminant alphaherpesviruses [20,21], we believe the virus circulating in these populations to be CvHV2. However future work in isolation or amplification of other less conserved areas of the genome should be carried out to help understand if the CvHV2 in Alaska is similar to the strains described in Scandinavia. Based on the serological prevalence over this time-frame in reindeer, the finding of alphaherpesvirus sequences by PCR, and the knowledge that these viruses establish life-long infections as any other herpesvirus, indicates that an alphaherpesvirus is endemic in the reindeer population and strongly suggests that CvHV2 is the agent circulating in Alaska. Extended sequencing of less conserved areas of the genome than the gB gene might enable us to verify if the CvHV2 strain in Alaska is indeed similar to Scandinavian isolates.

The higher frequency in older animals is consistent with previous studies. In Saskatchewan, it was found that the prevalence of herpesvirus antibody titers increased with age, with most seropositive animals being over three years old [18]. In Norway, the seroprevalence increased with age, weight and reindeer density (animals/km^2^) [13]. An increased frequency of seropositives in older animals is likely due to the increased time period that older animals have to become exposed and the age-cohort effect found in any enduring immunity. Animals, due to latency of the virus, are infected for life, having reactivations which boost production of antibodies. There was not a sex difference in the frequency of seropositivity even though males would be expected to have more interactions during the breeding season (fighting between males and breeding).

The single nucleotide difference between our PCR amplicons sequences and that of the Finnish CvHV2 strain (Salla-82) is identical to the substitution previously described in isolates obtained from Norwegian reindeer [17]. This leads to the question of whether the virus was imported with Siberian reindeer in the 1890s or if it has been present in the caribou population since they crossed the Bering land bridge over 13,000 years ago. Further studies, focusing on historical samples from the WAH, is needed to determine if the virus was circulating before the migratory pattern of this herd changed to cause more interaction with reindeer or if the caribou were exposed to the virus during these recent interactions. Although serological evidence of an alphaherpesvirus has been found in woodland caribou in Canada [18], the species of that virus is unknown and further evaluation is necessary to determine if it is the same virus as in Alaskan caribou and reindeer. If it is the same virus, this provides additional evidence that the virus has persisted in North America over several thousand years.

The impact of these viruses on the health of caribou and reindeer in Alaska is unknown. In Norway, CvHV2 has been shown to cause respiratory disease, abortions and keratoconjunctivitis [15,17]. It is, however, unknown if herpesvirus causes the keratoconjunctivitis reported in Alaskan reindeer or if the virus is one of many contributing factors in a multifactorial disease process.

Further research, including virus isolation from both reindeer and caribou is necessary to determine if the alphaherpesvirus circulating in reindeer and caribou in Alaska represent only one virus or strain and if it is indeed identical to the Scandinavian CvHV2. Studies on Siberian reindeer or reindeer imported from Siberia, having had less or no contact with caribou, may shed light on the origin of the virus in Alaska. Alphaherpesvirus infections in reindeer and caribou do not appear to induce high mortality or severe and obvious disease syndromes, but this is difficult to evaluate due to large and fluctuating herd sizes, remote areas and limited access to animals. However, being endemic and establishing latent infections, and having the potential to cause abortion, weak offspring, mucosal lesions as well as playing a part in the etiology of IKC, such infections may have greater impact on reindeer health and fitness than previously thought. This may be especially relevant to stress situations, reactivation of latent infections and shedding and transmission of virus within the herd, i.e. to calves and young animals.

## Conclusion

This study shows that Alaskan reindeer and Caribou are infected with an alphaherpesvirus. Based on sequence similarity, CvHV-2 is the most likely virus. Further studies should be conducted to determine the impact of this infection on the health of these animals.

## Methods

All methods were approved by the appropriate ethical committees (Alaska Department of Fish and Game, IACUC protocol number 09-12 and University of Alaska Fairbanks IACUC, protocol number 04-003).

### Sampling

**Figure 2:**
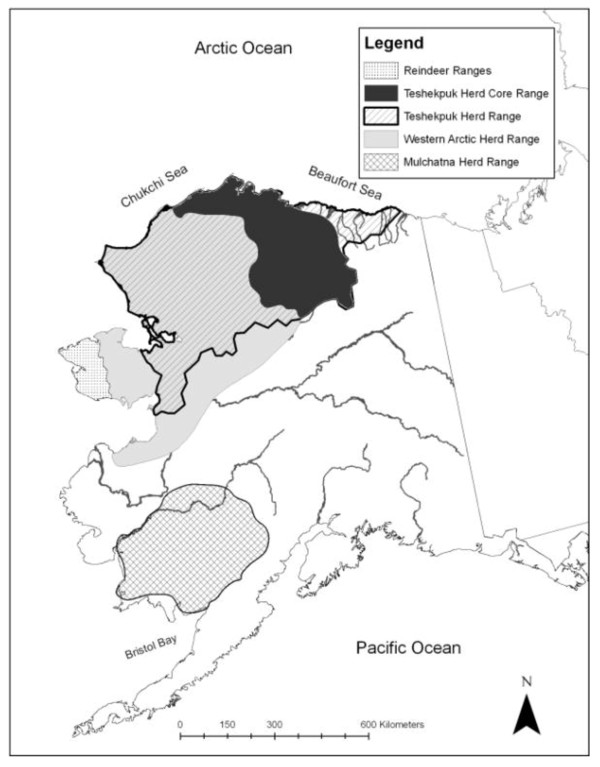
**Map of Alaska showing the range of the herds of caribou (*Rangifer tarandus granti*); Mulchatna, Teshekpuk and the Western Arctic caribou herds, and the semi-domesticated reindeer (*Rangifer tarandus tarandus*)**. The Western Arctic caribou herd has since 1996 expanded into the Seward Peninsula, creating problems for reindeer herding.

Serum samples from 292 reindeer were collected during the summers of 1988 through 2005 from the reindeer herd of the Davis family on the Seward Peninsula of Alaska (Figure 2). Each summer, reindeer were herded into a corral about 8 km north of Nome, Alaska (64°38'N, 165°20'W) for antler harvesting, tagging of calves and for herd health monitoring, including brucellosis vaccination and tuberculosis testing. Samples were stored at -62°C. In 2005, swab samples were collected from 40 calves from this herd, near Nome, Alaska. In 2007, ocular and nasal swab samples were collected from 30 apparently healthy reindeer calves near Wales, Alaska. Plasma and buffy coat samples were collected from three Alaskan caribou herds, the Mulchatna herd (n = 24, April 2009, N59°01'N, 156°27'W and 60°06'N, 160°53'W), the Teshekpuk herd (n = 34, June 2009, 70°24'N, 153°26'W) and the Western Arctic herd (WAH, n = 83, September 2009, 67°06'N 158°17'W), stored below -40°C until shipped on ice to our laboratory in Tromsø, Norway.

### Serology

Serum and plasma samples were tested using a commercial BoHV1 blocking ELISA (IBR gB blocking, LSI, Lissieu, France), which is based on glycoprotein B (gB) from BoHV1 as antigen, a highly conserved glycoprotein among ruminant alphaherpesviruses which enables the kit to detect antigenically related alphaherpesviruses [22]. Reindeer samples (n = 292) were tested in duplicate. Some caribou samples were tested in duplicate (n = 25) for validation and the remainder tested only in single wells. Samples with a competition percentage greater than 50% were considered positive, 45-50% inconclusive and less than 45%, negative, as previously validated for reindeer [22].

### Virus neutralization

A VNT including the viruses CvHV2, Cervid herpesvirus 1 (CvHV1), Elk herpesvirus (ElkHV) and BoHV1, was performed to determine which virus the animals were most likely exposed to and had produced antibodies against. This was done as previously described [13], choosing a subpanel of 10 caribou and 10 reindeer samples including samples with negative, weak positive and strong positive ELISA results. Serum was tested in two-fold dilutions up to 1:256, and neutralizing titers were calculated according to the Spearman-Kärber method [23] as the serum dilution necessary to neutralize the cytopathic effect (CPE) in 50% of the wells (effective dose 50%; ED50). A back titration of all viruses was performed to access TCID_50 _values for each of the viruses.

### PCR

DNA was extracted (DNeasy Blood & Tissue kit; Qiagen, Hilden, Germany) from swab samples collected from 30 apparently healthy calves in Wales, Alaska, 20 apparently healthy calves from the Davis reindeer herd and from buffy coats from 36 seropositive caribou from the WAH herd collected during Fall 2009. The buffy coat samples were extracted in pairs (100 μL of each sample, 200 μL total).

**Figure 3:**
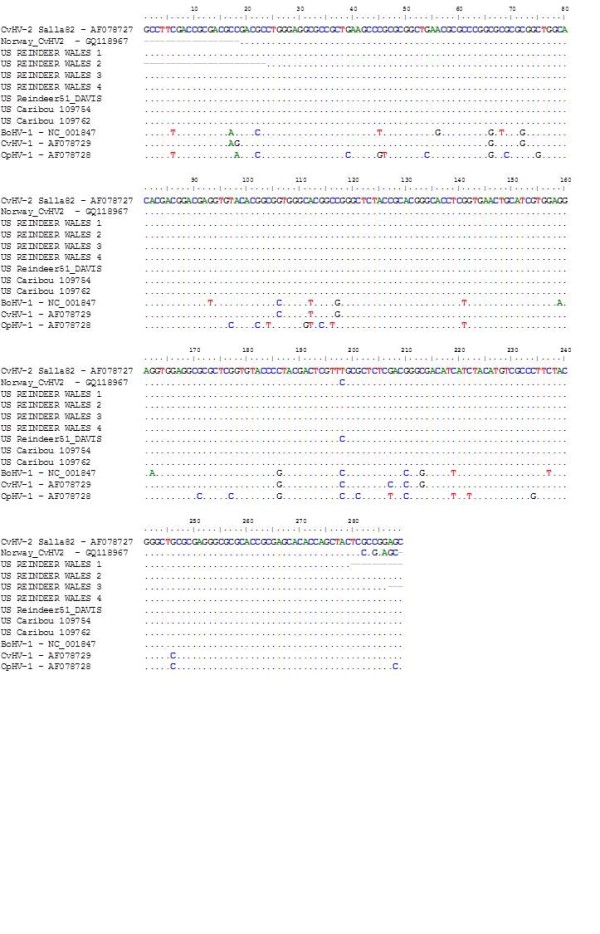
**Alignment of UL27 gene sequences of ruminant alphaherpesviruses obtained in this study and retrieved from GenBank**. Seven sequences were obtained in this study, 2 from caribou and 5 from reindeer and they are all identified by the letters US preceding its ID. All other sequences were obtained from Genebank and ascension numbers are shown in the sequence ID. Dots represent similarity to CvHV2 Salla 82, while displayed letters represent nucleotide differences.

Extracted DNA was amplified by a nested pan-alphaherpesvirus PCR as described previously [17,21], which has been widely used for identification of different ruminant alphaherpesviruses based on nucleotide differences in a highly conserved gene coding for glycoprotein B. CvHV2 (Finnish isolate, Salla82) was used as a positive control and sterile water as a negative control. Agarose gel (1,5%) electrophoresis was used to separate the PCR products and the gel was stained with ethidium bromide for visualization of DNA fragments. For amplicons of the expected size (294 base pairs), removal of primers and deoxynucleoside triphosphates, sequencing and sequencing alignment were done as previously described [17]. Figure 3 shows the alignment of the sequences obtained in this study with other ruminant alphaherpesvirus sequences available in GenBank.

### Statistical Analysis

After establishing the database in Excel^® ^for Windows, further statistical analyses were done in Stata 10/SE for Windows (StataCorp, College Station, TX. USA). A biweight kernel density estimation was done to evaluate the distribution of ELISA titers and to determine if the cut-off value of 50% was appropriate. A lowess (locally weighted scatter plot smoothing) was used to graph the effect of age on seroprevalence. Exposure frequencies per herd were calculated using the proportion command in Stata. A final logistic regression model was fitted to assess the adjusted associations of sex, age class (animals up to 1 year old being calves and above 1 year or older classified as adults), herd and year of sampling on serological status, seropositive or seronegative. The model was established using a backward selection procedure as previously described [24]. Model fit was assessed using the ROC procedure.

## List of abbreviations

CvHV2: Cervid herpes virus 2; BoHV1: Bovine herpesvirus 1; ElkHV: Elk herpesvirus; WAH: Western Arctic caribou herd; PCR: Polymerase chain reaction; CvHV1: Cervid herpes virus 1; VNT: Virus neutralization test; CPE: Cytopathic effect; ED50: Effective dose 50%; ELISA: Enzyme-linked immunosorbent assay.

## Authors' contributions

AE participated in study design, carried out the field sampling and data collection for Alaskan reindeer, performed laboratory analysis, analyzed data and drafted the manuscript. CN participated in design of the study, conducted and coordinated laboratory analysis, cooperated in the statistical analysis and participated in drafting the manuscript. GF participated in study design, coordinated field logistics, and contributed historical samples from reindeer. KB conceived of and facilitated inclusion of caribou in the study by participating in study design and coordinating and conducting field sampling of caribou. IN performed the ELISAs for Alaskan reindeer. ES was responsible for the statistical analysis. MT participated in study design and coordination and helped to draft the manuscript. All authors have critically revised the manuscript and read and approved the final manuscript.
